# Confirmatory Trials for Drugs Granted Conditional Approval by the Chinese National Medical Products Administration

**DOI:** 10.1001/jamahealthforum.2024.4601

**Published:** 2024-12-27

**Authors:** Yun Tian, Xiaoyong Liu, Xingyu Liu, Jinwei Zhang, Shuchen Hu, Caijun Yang, Yu Fang

**Affiliations:** 1 Department of Pharmacy Administration and Clinical Pharmacy, School of Pharmacy, Xi’an Jiaotong University, Xi’an, China; 2 Center for Drug Safety and Policy Research, Xi’an Jiaotong University, Xi’an, China; 3 Department of Pharmacy, Shaanxi Provincial Cancer Hospital, Xi’an, China

## Abstract

This cross-sectional study analyzes differences in pivotal and confirmatory trial characteristics for drugs granted conditional approval in China.

## Introduction

In 2018, the Chinese National Medical Products Administration (NMPA) established a conditional approval (CA) program, similar to the US accelerated approval program, which uses surrogate end points for drug indications in serious or life-threatening conditions. Numerous drugs have been approved under the CA program, mainly supported by pivotal trials.^1^ Limited evidence exists on the characteristics of confirmatory trials conducted after approval. Thus, we reviewed the confirmatory trials of all drugs receiving CA in China from January 1, 2018, to December 31, 2022, and compared their characteristics, including differences in design, with those of pivotal trials.

## Methods

In this cross-sectional study, we used publicly available NMPA data from ChinaDrugTrials.org and ClinicalTrials.gov to identify drug indications receiving CA. As the study did not constitute human participant research, local ethics review and informed consent were not required in accordance with the Common Rule. The study followed the STROBE reporting guideline.

We extracted all characteristics of the 2 trial types. Descriptive statistics were collected to characterize the indications and clinical trial features, with differences analyzed using χ^2^ tests for categorical variables and Wilcoxon rank sum tests for continuous variables. A 2-tailed *P* < .05 was considered significant. All analyses were performed using SPSS, version 26 (IBM Corporation).

## Results

The NMPA granted CA to 64 drugs for 85 indications. Due to nondisclosure of several confirmatory trials by the NMPA, 53 drugs with 67 indications were included in the final analysis (Table 1). Of these 67 indications, 38 (57%) were chemical and 40 (60%) novel or primary, and 36 drugs (54%) were domestically manufactured. Conditional approval was granted primarily for oncology indications (59 [88%]), and 39 indications (58%) were included in the medical insurance catalogs.

**Table 1.** Characteristics of 67 Indications Receiving National Medical Products Administration Conditional Approval, 2018-2022^a^

Characteristic	No. (%) of indications receiving conditional approval
Fast track	
Priority review	59 (88)
Breakthrough therapy	3 (4)
Drug class	
Chemical	38 (57)
Biologic	28 (42)
Chinese medicine	1 (1)
Approval type	
Novel	40 (60)
Supplemental	18 (27)
Unclear	9 (13)
Therapeutic area	
Solid tumor	42 (63)
Hemangioma	17 (25)
Nononcology	8 (12)
Manufacturing location	
Imported	31 (46)
Domestic	36 (54)
Medical insurance catalog^a^	
Yes	39 (58)
No	28 (42)

^a^ In China, drugs covered by medical insurance are categorized into class A and class B, which have different reimbursement rates. Class A drugs are reimbursed in full according to the regulations, while for class B drugs, individuals bear a certain percentage of the cost. All indications in the table that are included in the medical insurance catalog are class B medical insurance.

A total of 79 pivotal and 72 confirmatory trials supported the CA of the 67 indications. Confirmatory trials were mostly randomized (48 [67%] vs 29 pivotal trials [37%]; *P* < .001) and more likely to be double masked (23 [32%] vs 11 [14%]; *P* = .008) (Table 2). Progression-free survival as a primary end point was more common for confirmatory (31 [43%]) vs pivotal (13 [16%]) trials (*P* < .001). Overall survival (OS) was the least used end point in both trial types (8 [10%] vs 12 [17%]). Phase 3 was most common among confirmatory trials (47 [65%]) and phase 2 among pivotal trials (46 [58%]; *P* < .001). The median (IQR) time to completion of 66 confirmatory trials (excluding 6 with none reported) was 56 (40-73) months. By December 31, 2023, 12 of 21 (57%) trials did not meet the required completion time, including 2 completed after the deadline.

**Table 2.** Comparison of Pivotal and Confirmatory Trial Characteristics for Drugs Receiving Conditional Approval

Study characteristic	No. (%) of trials		*P* value
	Pivotal (n = 79)	Confirmatory (n = 72)	
No. enrolled,^a^ median (IQR)	130 (84-334)	202 (80-430)	.26
Randomized	29 (37)	48 (67)	<.001
Double masked	11 (14)	23 (32)	.008
Comparator			
Placebo	9 (11)	14 (19)	<.001
Active	14 (18)	25 (35)	
Placebo and active	2 (2)	5 (7)	
None	54 (68)	23 (32)	
Unclear^b^	NA	5 (7)	
Trial phase^c^			
1	16 (20)	6 (8)	<.001
2	46 (58)	17 (24)	
3	25 (32)	47 (65)	
4	NA	8 (11)	
Primary end point^d^			
ORR^e^	47 (59)	20 (28)	<.001
PFS	13 (16)	31 (43)	
OS	8 (10)	12 (17)	
Other	16 (20)	15 (21)	

Abbreviations: ORR, objective response rate; OS, overall survival; PFS, progression-free survival.

^a^ The number of people is based on the actual enrollment. For trial status currently recruiting or before recruiting, we used the trial number of expected enrollment as a reference.

^b^ These trials were set up with parallel control groups, but comparator information was unavailable in ClinicalTrials.gov or ChinaDrugTrials.org.

^c^ The trial phase may be 1 or 2I, or it may be 2 or 3, so the total percentage is more than 100.

^d^ Some primary end points were ORR and PFS or other (eg, event-free survival, time to progression), so the total percentage was more than 100.

^e^ Complete response and partial response for hematologic indications are also classified as ORR.

## Discussion

We found significant differences in the design and characteristics of pivotal and confirmatory trials for CA drugs in China, including randomization, masking, use of comparators, trial phase, and primary end points. These results differ from those observed in US studies, which found pivotal and confirmatory trials to have similar design characteristics.^2^

The primary end point is crucial for determining clinical benefit.^3^ In China, pivotal trials were more likely to use objective response rate while confirmatory trials favored progression-free survival as the primary end point, similarly to the US.^2^ The criterion standard of OS^4^ was underused in both pivotal and confirmatory trials. Due to poor correlation with OS,^5^ surrogate end points may not indicate a clinical benefit. Some studies^4,6^ found that nearly all accelerated approval withdrawals occurred for drugs whose confirmatory trials did not use OS as the primary end point. The NMPA should encourage companies to use OS as a primary end point in confirmatory trials, and physicians and patients should be aware of limited evidence of clinical benefits for CA drugs in China. This study used publicly available data spanning just 4 years, which limited the sample size and may have limited our ability to capture all pivotal and confirmatory trials.

## Notes

Supplement.
Data Sharing Statement
