# Supplementary material for: Confirmatory Trials for Drugs Granted Conditional Approval by the Chinese National Medical Products Administration
Source: JAMA Health Forum. 2024 Dec 27;5(12):e244601. doi: 10.1001/jamahealthforum.2024.4601 (PMC11681369; doi:10.1001/jamahealthforum.2024.4601)
Supplement: Supplement. — Data Sharing Statement [file jamahealthforum-e244601-s001.pdf]

Data Sharing Statement

Tian. Confirmatory Trials for Drugs Granted Conditional Approval by the Chinese National Medical Products Administration. *JAMA Health Forum*. Published December 27, 2024. doi:10.1001/jamahealthforum.2024.4601

Data
Data available: No
